# Living with a genetic, undiagnosed or rare disease: A longitudinal journalling study through the COVID‐19 pandemic

**DOI:** 10.1111/hex.13405

**Published:** 2022-02-05

**Authors:** Malia Byun, Hollie Feller, Monica Ferrie, Stephanie Best

**Affiliations:** ^1^ California Lutheran University California USA; ^2^ Genetic Support Network Victoria Melbourne Victoria Australia; ^3^ Australian Genomics Health Alliance Murdoch Childrens Research Institute Melbourne Australia; ^4^ Australian Institute of Health Innovation Sydney Australia

**Keywords:** chronic illness, COVID‐19, genetics, journalling, lived experience, rare disease, resilience

## Abstract

**Introduction:**

COVID‐19 changed the way we lived with uncertainty from the outset as the pandemic impacted every aspect of our lives from well‐being, socializing to accessing healthcare. For people in vulnerable populations, such as those with genetic, undiagnosed and rare disorders, the experience was heightened.

**Aim:**

The aim of this study was to identify how the rapidly changing COVID‐19 environment impacted the lives of the Genetic, Undiagnosed and Rare Disease community.

**Methods:**

From June 2020 to May 2021, we collected monthly open‐ended journals from people living in Australia with genetic, undiagnosed and rare disorders. Data analysis was deductive, using the Resilience Scale for Adults, and inductive using thematic analysis.

**Results:**

We recruited 29 people (average of *n* = 9.7 submissions each month). Responses changed over the year, with initial journals focusing on the importance of developing new structures for day‐to‐day lives, while later journals started to focus on mental well‐being. Throughout the project, participants reported challenges in accessing health and social care that was compounded by fear and concern over being exposed to the virus. Later journals highlight inconsistent messaging for vaccinations for this vulnerable community.

**Discussion/Conclusion:**

In parallel with the waves of the COVID‐19 pandemic, there need to be waves of targeted support for vulnerable communities. The first support wave needs to focus on facilitating the identification of new frameworks to structure day‐to‐day lives. A later second wave needs to focus on mental well‐being and coping with isolation, while consistent communication relating to health and social care throughout was essential.

**Patient/Public Contribution:**

This study was co‐designed, co‐led and analysed with a patient support network.

## 1 INTRODUCTION

Genetic, Undiagnosed and Rare Diseases (GUaRDs) individually present infrequently, but are collectively common. In Australia, ‘rare diseases’ affect approximately 6%–8% of the population, impacting an estimated 1,574,000–2,063,200 people and their families.^1^ People with GUaRDs are a vulnerable population, facing challenges in their search for a diagnosis, often referred to as the diagnostic odyssey, with multiple expensive and invasive investigations.^2^ In addition, their day‐to‐day lives are impacted by multisystemic chronic illness, frequently with acute exacerbations.^3^ Furthermore, those in the GUaRD community can face difficulty in connecting with peers and integrating seamlessly into the population whether through mainstream education or employment.^3^ The lived experiences of the GUaRD community commonly centre on one patient group for example, fragile X^4^ and epidermolysis bullosa^5^; however, it is important to understand the experiences of the whole community.^6^ As such, the Australian Federal Government has recognized the vulnerability and previous lack of focus on the GUaRD community and developed a National Strategic Action Plan with three pillars: (1) awareness and education, (2) care and support and (3) research and data aimed at promoting the ‘best possible health and wellbeing outcomes’ for this community.^7^ ^(p5)^

The COVID‐19 pandemic has impacted all Australians,^8^ though it has disproportionately affected vulnerable communities internationally with increased shielding and challenges with access to essential health and social care services.^9^, ^10^ The lived experiences of people with GUaRD, their carers and the support sector during this time of upheaval provided a unique opportunity to gather an understanding of how COVID‐19 has affected their lives (both positively and negatively) and how this will shape their needs in the future. When faced with unprecedented circumstances, how this vulnerable community would respond to the uncertainty of a global pandemic and enforced restrictions is not known. Resilience has been defined as ‘the process of adapting well in the face of adversity, trauma, tragedy, threats or even significant sources of stress’.^11^ ^(para4)^ However, resilience is not static and is known to exist on a continuum requiring a range of coping strategies to be deployed at different times.^12^ For the purposes of this study, we define resilience as the dynamic ability to maintain/restore relatively stable functioning, which will ebb and flow, when confronted with stressful life events and adversity.

The need for resilience is not confined to vulnerable communities. However, even before the pandemic, the GUaRD community had to draw on their personal strengths to overcome adversity to cope with the day‐to‐day challenges that present from living with a chronic illness.^13^ The use of coping strategies has been associated with better quality of life for those with chronic illness^14^ and their carers,^15^ though the nature of a crisis, for example, the cause of the crisis, duration and extent of resource loss, is known to influence the efficacy of the mechanisms used. Previous pandemics, such as Ebola and Severe Acute Respiratory Syndrome (SARS), provide some learning on chronic illness, resilience and crisis situations. Though not linked to chronic illness, the International Medical Corp^16^ reported an increase in mental health and psychosocial problems during the Ebola virus in Sierra Leone in 2014/15. In particular, they noted several factors linked to mental well‐being such as, fear, stress and isolation. During the SARS outbreak in Hong Kong in 2003, Lau et al.^17^ and others^18^ observed that the subjective well‐being of the elderly population was lowered, while young adults maintained their well‐being within normative levels.

Keeping a journal is a commonly used approach to data collection in health services research,^19^, ^20^ though typically addressed at symptom management.^21^, ^22^ The use of journalling allows the capture of day‐to‐day activities alongside emotion and self‐reflection.^23^ As a research method, journalling permits the collection of longitudinal experiences and allows participants to be at ease with their level of self‐disclosure.^23^ However, there appears to be a paucity of studies using journalling for data collection relating to people with a chronic illness that explore the lived experience, beyond immediate health presentations, with a methodological preference for semistructured interviews to gather lived experiences, for example.^24^, ^25^ By contrast, this study looks to use journal keeping as a mechanism to capture longitudinal changes in the lives of the GUaRD community.

The aim of this study was to investigate how the rapidly changing COVID‐19 environment influenced the lives of the GUaRDs community. In particular, we ask,

(1) How has the COVID‐19 pandemic impacted the well‐being and resilience of people in the GUaRD community and what coping mechanisms have they used?
(2) How has the COVID‐19 pandemic affected access to health and social care services?
(3) What lessons can be learnt for future health and social care service provision for people in the GUaRD community?

## 2 MATERIALS AND METHODS

### 2.1 Context

**Figure 1:**
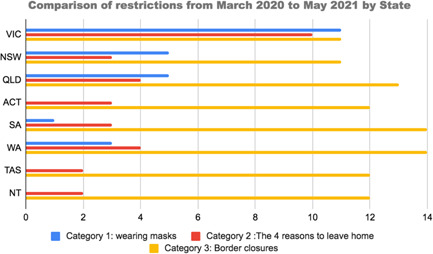
Comparison of COVID‐19 restrictions by state, March 2020 to March 2021. *Sources*: State Departments of Health and news agencies (detail in File S1)

The global COVID‐19 pandemic has been affecting countries worldwide at different times and with different intensities, with various governments enacting varying policies to manage the health of their populations. In Australia, COVID‐19 has affected the various states of Australia in different ways. Figure 1 shows how Victoria endured the most extensive restrictions with 9 months of mask wearing and 8 months of restrictions related to leaving the house. In contrast, the Northern Territories have experienced no mask wearing and only 2 months of restrictions related to leaving the house. Additionally, Victoria is the only state to have imposed an extra restriction, limiting residents to stay within 5 km of their home.

The Genetic Support Network Victoria (GSNV) (https://www.gsnv.org.au/), a statewide organization focused on supporting people living with GUaRD and those who support them, and Australian Genomics (https://www.australiangenomics.org.au/), a research funded network supporting implementation of genomic medicine collaborated to codesign and deliver the study.

### 2.2 Research design

This phenomenological, longitudinal, qualitative research study utilized open prompted journals to capture the lived experience of individuals with a GUaRD, their carers and support groups. Participants of the study were asked to keep a journal in any form they preferred (e.g., digital text, handwritten) in their preferred format (e.g., text, pictures) to share their lived experiences during the COVID‐19 pandemic.

### 2.3 Participants and recruitment

Individuals, older than 18 years of age, with a GUaRD, their carers or support group leaders living in Australia were eligible. Recruitment into the study was conducted through patient support group websites and their social media streams (e.g., Instagram). Potential participants were invited to contact the research team directly to obtain more information about the study. Participants who were interested were emailed a participant information sheet, written consent form, demographics form and the invitation to ask any questions. The recruitment period was through May 2020. Once participants returned the written consent and demographics form, they were considered as an enrolled participant for the study and sent the User Guide (File S2).

### 2.4 Data collection

Participant journals were open‐ended, and participants were free to share as much or as little as they preferred. Journals were collected via email each month from July 2020 until May 2021. Participants were informed each month, by email from the study admin lead, that the journals were due, in any format. There was no obligation to send in a journal and participants could opt out of the study whenever they wished. Regular study updates were provided via the GSNV newsletter. Each participant was given a number, and their journals including visual submissions were filed via a Virtual Private Network by month and participant number.

### 2.5 Data analysis

We adopted a five‐step approach to the longitudinal data analysis.^26^ (1) *Consider the analysis approach*: The team discussed the potential for different approaches to analysis as the journals were being submitted over the first few months. Initially, we selected a deductive approach to help identify findings in relation to resilience theory.^27^ (2) *Setting up an analytic roadmap*: To respond to the research questions, data were initially analysed using a resilience scale. Numerous resilience scales exist including some relating to genetic conditions (e.g., the Psychological Adaptation Scale).^28^ However, these frameworks did not take into account the impact of unforeseen events for example, an international health crisis. As such, we looked to the broader resilience literature and identified the Resilience Scale for Adults (RSA).^29^ This validated scale has been used in a range of settings and found to be responsive to users' experiences of resilience in crisis environments.^30^, ^31^ The RSA has five constructs, which include the perception of self, social competence, structured style, family cohesion and social resources (Table 1).

**Table 1:** Adapted Resilience Scale for Adults (RSA)^26^ applied in context

**RSA code**	**RSA descriptor**	**Description in context (third person)**	**Description (internally imposed)**	**Description (externally imposed)**
Structured style	‘I sustain my daily rules even in difficult situations’	Daily rules/routines can be sustained, even in difficult situations	The choice to continue (or discontinue) daily rules/routines is made by the individual	The continuation (or discontinuation) of daily rules/routines is influenced by an external factor (e.g., family members, doctors, etc.), not the individual
The perception of self	‘I know if I continue, I will succeed’	The individual believes that they can continue on their path and achieve success	The desire to continue and achieve success is mentally driven by the individual	The desire to continue and achieve success is influenced by an external factor (e.g., family member, therapist, etc.)
Family cohesion	‘Even in difficult situations, my family is optimistic’	Family members remain optimistic, even in difficult situations	N/A	N/A
Social resources	‘There is always someone who helps me when I'm in need’	There is always someone who can help, when and where help is needed	N/A	N/A
Social competence	‘I can establish friendly relationships easily’	The individual can easily establish a range of relationships and is comfortable in social settings	The individual makes the effort to establish relationships and chooses to initiate social interactions	External factors (e.g., carers, therapists, etc.) influence the individual's decisions to establish relationships and initiate social interactions

### 2.6 Familiarization and coding

Journals were cleared of any personal identifiers and replaced with pronouns or pseudonyms to maintain confidentiality among the participants. Each participant was given an interviewee number for example, 1, 2, 3 and so forth location by state (QLD—Queensland, NSW—New South Wales, VIC—Victoria, WA—Western Australia), their role (e.g., carer) and age group. Each RSA division was used as a code for the data. The first month's journal submissions were coded independently (M. B. and S. B.) before gathering together to discuss how entries were categorized. On familiarizing ourselves with the journals, the coders (M. B. and S. B.) identified additional potential codes in the journals. The need to evolve the approach to data analysis is common in longitudinal qualitative research,^27^ and we supplemented the deductive data analysis using thematic analysis.^32^ Additional thematic codes included the direct impact of COVID‐19 on health and social care services, turning points and coping strategies related to the pandemic (Table 2). The coding was then completed by one author (M. B.) with fortnightly meetings to continue to discuss and resolve challenging coding. Visual submissions (photographs) were tied (M. B. and S. B.) to RSA codes or inductive codes where possible. (4) *Describing cross‐sectional data*: Data analysis began as cross‐sectional analysis, describing the findings month by month. Once over 6 months of data had been collected, the research team regularly reviewed the monthly analysis to identify potential longitudinal patterns in the coding.^33^ Finally, (5) *Exploring longitudinal data*: After all the data had been collected, we adopted a trajectory approach to analyse our findings, to identify how experiences had changed over time.^34^

**Table 2:** Additional thematic codes

**Thematic codes**	**Descriptor**
Direct impact of COVID‐19 on health and social care services	Changes in access, experience or protocol of health and social care services as a direct result of COVID‐19
Turning points of pandemic experiences	Positive and negative changes in: the pandemic accessing peer support accessing healthcare services
Coping strategies	mechanisms utilized to cope with the pandemic including lockdowns and restrictions coping with the impact of GUaRD during the pandemic

## 3 RESULTS

First, we present the characteristics of the participants and their journals before outlining the findings from the written text journals. Themes from the journals relate to the RSA and additional thematic analysis (see File S3 for additional data from the journals supporting the RSA and thematic analysis).

### 3.1 Response rates

**Figure 2:**
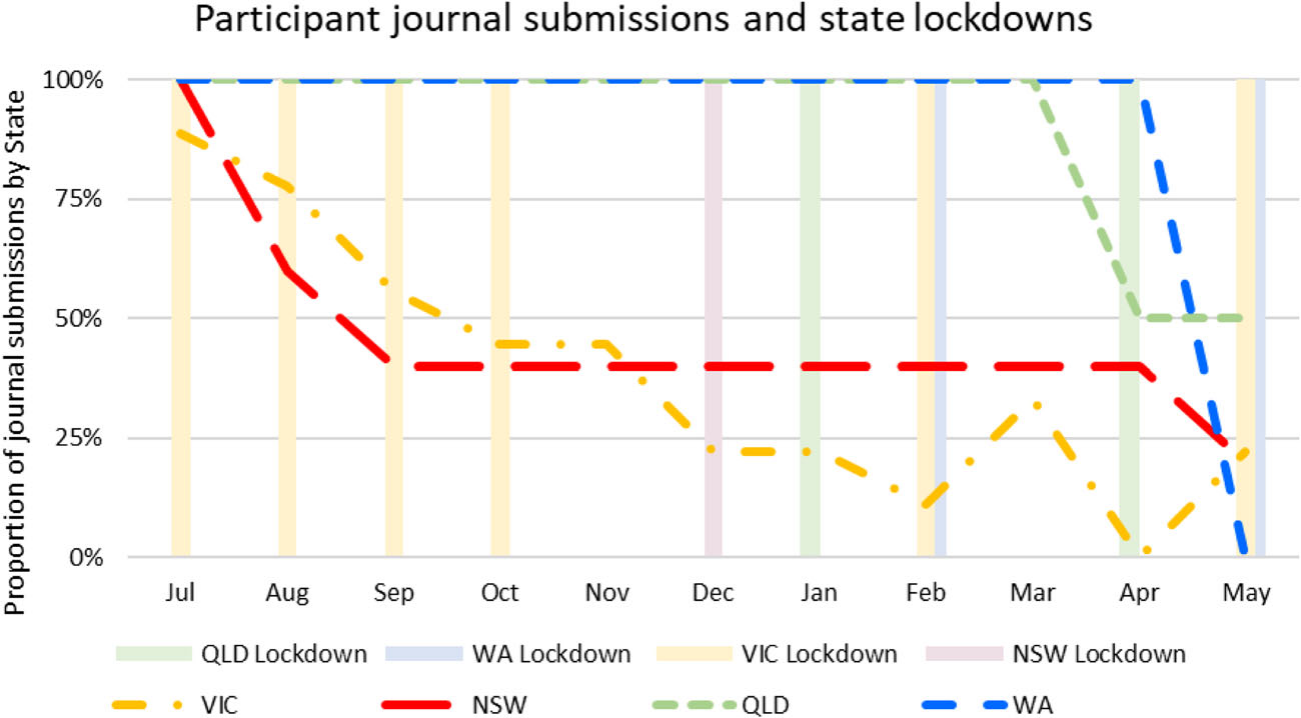
Journal submissions by month

In total, 27 people expressed an interest in taking part in the journalling project, completing the participant ‘demographics’ survey. Seventeen journals were received at the first submission date in July 2020, and these 17 participants continued to contribute over the year. We subsequently received an average of 9.7 journals each month (Figure 2).

### 3.2 Characteristics of participants and journal submissions

Most participants were aged 46–59 years (*n* = 7), with six people aged 18–29 years, three people aged 30–45 years and one between 60 and 75 years of age. The majority were based in metro locations (*n* = 14), with eight people based in Victoria, six in New South Wales, two in Queensland and one in Western Australia. People played more than one role in the community, with most having a GUaRD (*n* = 11); seven people were carers and six were part of the peer support sector for people with GUaRD and/or their carers. Contributions dropped off over the year for both carers and for people with GUaRD. More people withdrew from the state of Victoria, which experienced the most enduring lockdown in Australia at this time.

### 3.3 Characteristics of journals

Most participants (*n* = 10/17) solely used text using a word processor facility to record their journal. One participant hand wrote their journal and sent in photographs of their writing. Several participants (*n* = 5/17) submitted mixed media journals combining photographs, graphics and text. One participant sang their submission and sent it in as a video. The written journals varied in length from a few lines to several pages, with an average length of 5.5 pages, which remained fairly consistent over time.

### 3.4 Themes

There was some variation in the themes identified by age group, experience and state. The experiences of carers were, at times, different from the experiences of people with GUaRD, and are reflected in the quotes across the themes. Additionally, age could influence responses; for example, younger participants tended to focus more on their personal life and schooling:

I had to go into school for the exams, and I got to see some of my friends who I haven't seen in months. (Participant 24, NSW, Individual, age 18–29, September 2020)

However, older participants were more commonly carers and reported wider experiences including healthcare, family dynamics, sustainability, politics and finances:

I was very happy to hear that pollution in some busy world cities decreased during the lockdown and that the Himalayan peaks were visible from parts of India for first time in decades. The communal singing from the balconies in Europe was lovely to witness on the TV and I heard that the water in the Venice canals was clearer, and you could see the bottom. As people have had a taste of lower pollution, I certainly hope there is more investment in renewable energy. (Participant 9, NSW, Individual, age 46–59, July 2020)

Additionally, older participants were more likely to use a wider range of journal recording methods. The pandemic experience varied by state, with Victoria experiencing a greater incidence of COVID‐19 (and Western Australia had the least exposure). As a result, participants from Victoria reported more COVID lockdown than other states, although all states experienced travel restrictions and the need to access services by telehealth.

#### 3.4.1 RSA code: Structured style

The theme of structure was richly saturated at the outset of the project (July 2020 to September 2020), with many participants reporting the ways in which their usual routine was upset:

The challenge now was to try and get back a sense of routine amongst the unpredictability of being in lockdown. (Participant 1, VIC, Carer/Support Sector, age 46–59, July 2020).

I have a two year old son so it has been hard to keep him entertained while trying to limit his screen time where normally we are very active and enjoy going to the park and to his gymnastics class. (Participant 11, NSW, age 18–29, July 2020)

Additionally, some, though not all, reported their drive to carve out a new daily structure to frame their days:

We set an agreed list of tasks and activities for him to do each day. In his diary we write: 9‐10am computer; 10‐11am reading; 11‐11:30am empty dishwasher, jigsaw; 1pm go for a walk; 1:30pm lunch; 2‐3pm playstation; 3‐4pm reading. And on it went, adding different chores and mixing the routine around each day. This scheduling in the diary helps give him the routine and predictability he craves. (Participant 1, VIC, Carer/Support Sector, age 46–59, July 2020)

With [my son] and [daughter] both studying we had a good schedule for classwork, breaks, lunch outside for some fresh air and vitamin D, and some afternoon walks around the beach. (Participant 29, NSW, age 46–59, July 2020)

Towards the middle and the end of the study period (from October 2020 onwards), relatively few journals were coded to the RSA structure theme.

#### 3.4.2 RSA code: The perception of self

Coding related to perception of self (i.e., I know if I continue, I will succeed) peaked around the middle of the submission timeline (October 2020 to December 2020). Participants struggled with their mental well‐being through the enduring restrictions and lockdowns due to isolation, lack of independence and/or lack of social interaction:

Feeling pretty defeated (it's was a LOT of effort for what should have been relatively straight forward) but tomorrow is always another day. (Participant 21, QLD, age 18–29, October 2020)

There was hesitation in attempting to continue on, implying that a sense of self was also challenged:

Self‐doubt strikes that chord again that sings ‘What makes this attempt any different? Failure is your fate. Deceit and cowering away is your destiny’. So, I haven't committed to anything yet. (Participant 21, QLD, age 18–29, November 2020)

#### 3.4.3 RSA code: Family cohesion

Responses relating to family cohesion remained constant throughout the project. Journal submissions were mixed between negative and positive impacts. Initially, participants reported difficulties with not seeing family:

doing it without your ‘tribe’ is heart breaking, soul crushing and terrifying. (Participant 7, VIC, Carer, age 30–45, July 2020)

As the restrictions in many states continued, there were positive and negative experiences impacting on family life:

I sent an email to CEO and service manager [Elle] to say I'll take [Marvin] home for social leave. Before I start the ball rolling I asked [Maya] if she would be happy to help out if her Dad came home. She was very positive about it. (Participant 28, WA, Carer, age 60–75, July 2020)

Emotions in our house seem a bit more intense over the last few weeks, the kids are both a bit touchy and have been bickering a little bit which is unusual for them. [My son and daughter] are very close and get on really well, I'm very lucky that I almost never have to deal with sibling tensions like other families do. We are all pretty good communicators so a quick chat about being a bit more understanding of the stresses in the house and each other nipped the issue fairly quickly. (Participant 29, NSW, Carer/Support Sector, age 46–59, August 2020)

#### 3.4.4 RSA code: Social resources

Here, social resources construct reflects that there is always someone who can help when a person needs this support. The social resources construct was less richly saturated in comparison to some other RSA themes. However, it played an important role across the pandemic as people sought out health‐oriented resources:

I have arranged extra support workers for [Marvin] and arranged for FaceTime with [Marvin] while NDIS workers are with [him]. (Participant 28, WA, Carer, age 60–75)

Wider resources, including access to peer support, were challenging for people to access, except virtually:

He will have the support of an Education Assistant and an Auslan interpreter (when required) during classes. (Participant 1, VIC, Carer/Support Sector, age 46–59, July 2020)

#### 3.4.5 RSA code: Social competency

Social competency, as an RSA code, refers to the ability to be able to strike interactive relationships with ease. The limitations in social interactions imposed by the pandemic had a mixed impact on the journal participants. For some, the shift to online communication removed some of the complexities (such as social anxiety, mobility, sensory disabilities) usually involved in interacting with others:

Emotionally I feel quite good this evening after the online socializing. I've found zoom sessions with [a support group] or my friends more broadly to be fantastic motivators and energizers for me, even with slightly dodgy internet. (Participant 21, QLD, Individual, age 18–29, January 2021)

I have been able to meet new people through the online world who I wouldn't otherwise have known and I am thankful for these opportunities. Being able to access so many free activities all from the comfort of my home has made life so much easier for me (Participant 23, VIC, Individual/Support Sector, age 18–29, September 2020)

For others, the move to technology‐based communication was more difficult, for example, with the shift to telehealth consultations. Some members of the community required support to communicate in the technology‐based environment:

Lockdown 2 has brought new challenges for [Shane's] independence as well, as telehealth has replaced face to face appointments. While we have been making some progress in his transition to being an independent health care recipient, it has been disappointing to find that under telehealth, his independence has receded because his accessibility needs in this online setting have not been considered. (Participant 1, carer/support sector, age 46–59, VIC, September 2020)

Lockdown led to restrictions in what people could do, and some, often younger participants, felt an inertia. They often submitted very limited journals each month, reporting that they had little to feed back on, suggesting a withdrawal from socializing from the usual busy activity of young adult life:

‘So, I haven't been doing much’ and ‘there is not much to do’. (Participant 6, VIC, Individual, age 18–29, August 2020)

#### 3.4.6 Theme: Direct impact of COVID‐19 on health and social care services

The pandemic had direct impacts on participants. Some challenges became apparent early on, for example, as healthcare facilities rapidly restricted the facilities available, participants needing regular care or specialized care struggled to access them:

The doctors are still trying to get her back to the Gastro clinic at BHH but they are still delaying appointments due to covid. (Participant 27, VIC, Carer, age 46–59, July 2020)

Delaying routine healthcare appointments for this population, either through lack of availability or consumer preference, leads to the risk of resulting in ongoing health issues longer term. Additional services for example, therapies and social care were also curtailed as service providers withdrew service provision to minimize transmission risks for staff and families:

Professional driving lessons were put on hold from the brain rehab OT driving instructor. (Participant 2, NSW, Carer/Support Sector, age 46–59, July 2020)

Similarly, peer support functions and training services withdrew provisions:

One of the most difficult and truly disappointing things has been that ANE research and our inaugural ANE conference has had to be put on hold directly because of Covid19. (Participant 2, NSW, Carer/Support Sector, age 46–59, August 2020)

Unfortunately, he missed 6 weeks of TAFE due to COVID‐19. This was not the experience we had hoped for him at TAFE. (Participant 1, VIC, Carer/Support Sector, age 46–59, July 2020)

Some participants proactively chose to stop their personal social support services to reduce the potential for transmission of the virus:

During the virus, because of the genetic condition I have and other health conditions I have, I have had to self‐isolate from day 0. (Participant 6, VIC, Individual, age 18–29, VIC, July 2020)

Later on, participants were conflicted over whether to have the vaccine or not:

Within our [EDS] Facebook communities, people are having heating arguments about whether or not EDS patients should have the vaccine. Many of us react badly to other vaccines with resulting increases in autoimmune issues, so we cannot be sure that we can have this vaccine without major reactions and increased disease burden… The uncertainty about the consequences of us having or not having the vaccine are very worrying. (Participant 27, VIC, Carer, age 46–59, February 2021)

#### 3.4.7 Theme: Turning points

Turning points, as described in Table 2, referred to where participants experienced a significant event or decision made as a result of the pandemic, for example, in accessing healthcare services or peer support and so forth. The COVID‐19 pandemic has been challenging for people in the GUaRD community. This participant exemplifies the experience of carers who have had to take the initiative and make difficult decisions to ensure the safety of the person with GUaRD since the onset of the pandemic:

Emotionally for myself I am feeling very drained and feeling very conflicted and guilty about the decisions I'm having to make for [my daughter] this year. (Participant 29, NSW, Carer and Support Sector, age 46–59, August 2020

Several turning points were identified in the journals, including the imposition of restrictions:

As stage 4 restrictions have loomed over Victoria, I am half‐way through my course via online learning (Participant 6, VIC, Individual, age 18–29, August 2020)

Conversely, opening up was not always welcomed:

Personally, work is busy and we are anticipating a ‘2nd wave’ at work, the hospital itself and the people in the community are taking it more seriously—or getting use to it—this is making it easier to manage when everyone is following the rules, we are waiting in anticipation of things getting worse (Participant 20, age 18–29, QLD, Individual/Support Sector, August 2020)

#### 3.4.8 Theme: Coping strategies

**Figure 3:**
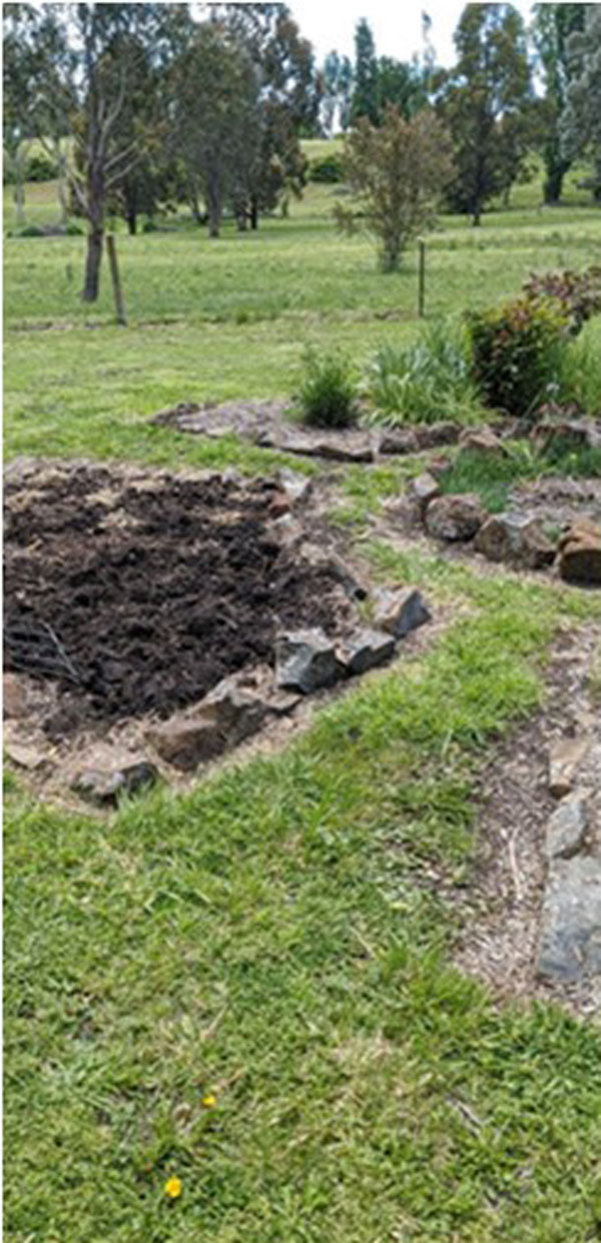
Work on the garden as a coping strategy. Reproduced with permission from Participant 2

We defined coping strategies in this study as the mechanisms that participants used to help manage the impacts of the pandemic (such as lockdowns) and/or the impact of GUaRD through the pandemic. Coping strategies varied and were often supported by a visual submission. For example, at home (Figure 3):

‘I did maintenance on my garden and laid down new mulch and created some soft borders using tree branches that I had cut down’… ‘I did outdoor runs and home exercise classes using zoom instructions offered by my gym’. (Participant 9, NSW, Individual, age 46–59, July 2020)

I've spent more time this year in preparing a veggie patch in my gardening. With the grandkids, we planted seeds and then when they sprouted, transferred them to pots and eventually the garden. I've been seeing so many other people during lockdown and since, turning their hands to ‘home grown’…We live in scary times and I guess it feels like a built in safety measure that we can, with enough time and effort, be self‐sufficient in some way. Plus, it has truly been fun doing this with the kids. (Participant 2, NSW, Carer/Support Sector, age 46–59, November 2020).

**Figure 4:**
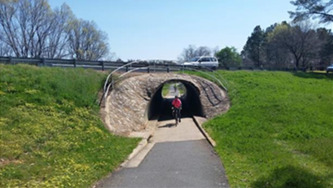
Cycling as a leisure activity coping strategy. Reproduced with permission from Participant 9

Various other leisure activities also provided a mechanism to get out and enjoy the local community facilities (Figure 4).

… I do enjoy the small trips I do close to home. I have had a few small walks with different girlfriends and I had a trip to Wollongong with my husband recently. (Participant 9, NSW, Individual, age 46–59, September 2020)

Recently I was lucky to have spent a few days holidaying in Canberra and the weather was very pleasant. My husband and I enjoyed some cycling, galleries and the tulip displays spread about Canberra… Due to pandemic restrictions, the 2020 Floriade Festival was not in its usual fixed location. Instead, one million bulbs and annuals were planted by the Floriade horticulture team and Canberra community groups, creating a tulip trail throughout the city and suburbs. (Participant 9, NSW, Individual, age 46–59, October 2020)

## 4 DISCUSSION

Our findings demonstrate how the COVID‐19 pandemic impacted the lives of the GUaRD community. We identified themes from the RSA throughout the year, though experiences for people of varying ages, locations and roles varied. Differing themes were more apparent at varying stages; for example, the need for structure was initially a very strong theme early in the pandemic, though not later once participants had re‐established a routine. The journals suggest that the first line of support that many people in the GUaRD community may need is guidance on refinding an order to their day that will fit with the uncertainty of living through a crisis period.

### 4.1 System supports for vulnerable groups

Isolation and fear of contracting COVID‐19 can lead to loneliness and chronic self‐isolation in all populations,^35^ though exacerbated in vulnerable communities.^9^, ^10^ Structure is recognized as a key component of resilience,^29^ and while some participants described how they generated a new framework for their days, not all did. Some participants may not have been aware of the potential benefit of carving out a new structure for their day‐to‐day life. In line with previous studies, mental well‐being was a strong theme in the journals.^17^ However, the importance of mental well‐being and resilience did not feature initially for our participants and began to feed into journals later in the pandemic as some people started to feel the loss of their independence. This may be surprising as many people in the GUaRD community were isolated before the pandemic; however, the ongoing fear and chaos of the COVID‐19 virus appeared to exacerbate this feeling. Alongside mental well‐being, there was a sense of inertia that impacted on perception of self, particularly amongst younger participants. This runs counter to previous findings that reported lower subjective well‐being amongst the elderly population during a pandemic.^18^ This finding suggests the need for peri‐ and postcrisis event mental health and well‐being services across the population.

There was no relief for carers, who, although often stoic, reported fatigue and frustration with the ongoing and unpredictable nature of the global pandemic impacting locally. Participants reported some coping strategies within the limitations of lockdown to alleviate rather than resolve the pressures that they were experiencing: some took short breaks to the beach, as restrictions permitted, while others took to their gardens. The visual submissions commonly reinforced the coping strategies used. Identifying potential coping strategies was essential to help sustain quality of life for both carers^15^ and people with GUaRD.^14^ In the social competency, we see peer support groups played a key role with online sessions.

Access to health and social care services, particularly early in the pandemic, was severely restricted. At times, this hiatus was due to service providers withdrawing both hospital‐ and community‐based services, while in some cases, participants chose to withdraw from formal care to lower the risk of virus transmission. With either scenario, there is concern about the impact that this lack of care may have on longer‐term physical and mental health for vulnerable groups.^8^ The move to online services provided mixed experiences, with challenges with technology and connecting with health professionals.

Participants recorded a varied picture of health and social care information provision across the year. Most recently, this is typified by the discussion around vaccinations. Vaccine hesitancy in Australia is already higher than in many other countries.^36^ Confused messages, misinformation, lack of information, fear and worry featured in journals as participants were unsure whether people with a GUaRD should access the vaccine due to their underlying condition. And if so, which one? The potential short‐ and long‐term impacts of vaccination are particularly unclear for people with immunocompromising comorbidities. The need to provide clarity and consistency in health information is essential for vulnerable groups.

### 4.2 Lessons for future crisis events

These findings are significant and provide lessons for future crisis events. Much as there have been waves of the COVID‐19 virus, our participants identify that there need to be waves of different support offered at different times when people in the GUaRD community need it. The first support wave needs to be focused around helping the community adjust and recognize the importance of finding a new structure for their lives. This is likely to be an iterative process that the GUaRD community needs to be aware of. The subsequent support wave needs to include mental well‐being support and finding a sense of self. While our journals suggest that this support is not needed immediately as a crisis occurs, as people are coping with the immediate effects of the pandemic and working out how it impacts them, it is clearly required a few months into the crisis. Our data also clearly indicate the need to support the younger population. Previous literature also suggests that this support will need to be continued once the crisis is alleviated.^18^ As the duration of the pandemic progresses, vulnerable populations need support in either finding their own coping strategies or where they can access either peer or formal support. Finally, the significance of the consistency of provision of health and social care information (including education) is paramount for this population. Throughout the crisis, there is a need for accurate information and signposting to health advice and also wider social support. Building confidence in vaccines is vital for all population groups in Australia,^37^ but especially so for this vulnerable group.

Our study reflected the experiences of people from the Genetic Undiagnosed and Rare Disease community living through a year of COVID‐19. Before the pandemic, this population was accustomed to overcoming challenges in accessing care and in day‐to‐day life.^38^ As such, this community, both carers and people with GUaRD, was experienced in resilience and therefore we adopted this lens to interrogate the journals.

Journalling as a research methodology offers both strengths and limitations. Participants are in control, with freedom to express whatever is of relevance to them in a mode that suits them both emotionally and physically. They can also decide what they are comfortable sharing and what, on reflection, they would prefer to keep private. It was clear that participants were fatigued towards the end of the study; however, we are unable to attribute this directly to either the journalling methodology or the pandemic. While there are benefits in empowering the participant, many of these features may be frustrating to the researcher, who may wish to delve deeper and be in control of data collection.^27^ These concerns could be mitigated with follow‐up interviews or focus groups to discuss the themes identified in the journals. Journalling provides an opportunity to gather longitudinal data, which aligns with capturing experiences during an ongoing global event. We started this project in June 2021, so missed experiences from the very early months of the COVID‐19 pandemic. However, the study design ensured that we were able to gather the longitudinal experiences of this vulnerable group. Participation was voluntary and although we encouraged journal submissions in any format and length, due to the nature of journal writing, it is likely that we had a bias towards higher educated participants.

## 5 CONCLUSION

This study supports the National Strategic Action Plan^7^ in promoting awareness of the impact of GUaRD and has shown that the lives of the GUaRD community have been severely impacted by the COVID‐19 pandemic. Timely support waves are required to ensure that this vulnerable community receives appropriate support when they need it including a first wave providing support to develop a day‐to‐day routine, consistency in health information and later, mental well‐being guidance and direction. In line with these findings, further work is required to design, develop and test mechanisms to support people in the GUaRD community during challenging times.

## CONFLICT OF INTERESTS

The author declares that there is no conflicts of interest.

## ETHICS STATEMENT

Ethical oversight was provided by the Royal Childrens Hospital, Melbourne, Human Research Ethics Committee, with approval received on 15 May 2020.

## Supporting information

Supporting Information.

Supporting information.

Supporting information.

## Data Availability

The data for this study are not publicly available due to privacy and ethical restrictions.
