# Supplementary material for: Living with a genetic, undiagnosed or rare disease: A longitudinal journalling study through the COVID‐19 pandemic
Source: Health Expect. 2022 Feb 5;25(5):2223–34. doi: 10.1111/hex.13405 (PMC9111564; doi:10.1111/hex.13405)
Supplement: Supplementary file 1 — Supporting Information. [file HEX-25--s001.docx]

**Supplementary file S1**

March 2020 - March 2021 Covid 19 DATA

References for excel spread sheet

General Border restrictions

<https://amp.abc.net.au/article/13047690>

<https://www.aljazeera.com/amp/news/2020/11/23/border-between-two-biggest-australian-states-reopens>

<https://www.abc.net.au/news/2020-12-31/border-restrictions-in-each-state-territory-for-nsw-and-victoria/13024468>

<https://www.abc.net.au/news/2021-01-21/border-restrictions-covid-qld-nsw-vic-sa-tas-act-wa-explained/13079000>

<https://www.abc.net.au/news/2021-02-12/victorian-outbreak-australia-reacts-where-you-can-travel/13150852#TAS>

<https://www.mediastatements.wa.gov.au/Pages/McGowan/2020/03/New-border-controls-to-help-protect-Western-Australia.aspx>

<https://www.abc.net.au/news/2020-05-09/how-travel-will-resume-around-australia-coronavirus-restrictions/12231242>

Mask info

<https://amp.abc.net.au/article/12641758>

<https://www.finder.com.au/face-mask-rules-australia>

Northern Territory

<https://www.abc.net.au/news/2021-01-19/nt-face-masks-at-darwin-airport-new-air-crew-protocols/13069474>

<https://amp.abc.net.au/article/12736560>

<https://newsroom.nt.gov.au/mediaRelease/32113>

<https://newsroom.nt.gov.au/mediaRelease/33193>

<https://newsroom.nt.gov.au/mediaRelease/33205>

<https://newsroom.nt.gov.au/mediaRelease/33205>

<https://www.mediastatements.wa.gov.au/Pages/McGowan/2020/05/Phase-3-of-COVID-19-roadmap-to-commence-from-Saturday-June-6.aspx>

NSW

<https://www.nsw.gov.au/media-releases/new-covid-19-restrictions-begin-as-schools-move-towards-online-learning>

<https://www.dhhs.vic.gov.au/coronavirus-update-Victoria-18-December-2020>

<https://www.nsw.gov.au/media-releases/health-advice-update-on-masks>

<https://www.nsw.gov.au/media-releases/update-on-covid-19-restrictions>

<https://www.nsw.gov.au/news/nsw-to-ease-covid-19-restrictions-from-friday-15-may>

<https://www.nsw.gov.au/news/new-covid-19-restrictions-place-across-nsw>

<https://www.nsw.gov.au/media-releases/restrictions-christmas-2020>

<https://www.nsw.gov.au/news/new-covid-19-restrictions-place-across-nsw>

health.nsw.gov.au/news/Pages/20210212_04.aspx

WA

<https://www.uwa.edu.au/news/article/2020/july/expert-warns-west-australians-not-to-get-complacent>

<https://www.perthnow.com.au/news/coronavirus/coronavirus-crisis-was-new-face-mask-rules-explained-ng-b881783936z.amp>

<https://www.mediastatements.wa.gov.au/Pages/McGowan/2020/03/Important-new-COVID-19-measures-come-into-effect-.aspx>

<https://www.abc.net.au/news/2020-11-18/sa-ordered-into-major-lockdowns-amid-coronavirus-outbreak/12894666>

<https://www.mediastatements.wa.gov.au/Pages/McGowan/2020/04/Cautious-easing-of-restrictions-thanks-to-WAs-COVID-19-progress.aspx>

<https://www.mediastatements.wa.gov.au/Pages/McGowan/2020/05/The-WA-roadmap-for-easing-COVID-19-restrictions.aspx>

<https://www.mediastatements.wa.gov.au/Pages/McGowan/2020/06/WA-ready-for-the-next-phase-of-the-COVID-19-roadmap.aspx>

<https://www.abc.net.au/news/2021-01-30/wa-to-relax-hard-border-with-qld-and-victoria/13105848>

ACT

<https://www.health.gov.au/news/acting-chief-medical-officer-press-conference-about-covid-19-on-20-december-2020>

QLD

<https://concreteplayground.com/brisbane/travel-leisure/face-masks-will-no-longer-be-mandatory-in-greater-brisbane-from-january-22>

<https://www.abc.net.au/news/2020-08-24/coronavirus-queensland-covid-19-restrictions-masks-brisbane/12588552>

<https://statements.qld.gov.au/statements/89738>

<https://statements.qld.gov.au/statements/89800>

<https://statements.qld.gov.au/statements/89931>

<https://statements.qld.gov.au/statements/89582>

SA

<https://7news.com.au/lifestyle/health-wellbeing/sa-covid-restrictions-lockdown-for-adelaide-essential-workers-only-no-exercise-c-1602556>

<https://www.abc.net.au/news/2020-11-18/sa-ordered-into-major-lockdowns-amid-coronavirus-outbreak/12894666>

<https://www.covid-19.sa.gov.au/latest-news/covid-restrictions-to-ease-from-monday>.

<https://7news.com.au/lifestyle/health-wellbeing/coronavirus-australia-sa-brings-stage-two-of-restrictions-easing-to-june-1-c-1058203>

<https://www.premier.sa.gov.au/news/media-releases/news/fast-tracking-economic-recovery>

<https://www.miragenews.com/updates-to-covid-19-directions-for-18-february-515430/>

<https://www.abc.net.au/news/2021-01-12/sa-keeps-nsw-brisbane-coronavirus-border-restrictions-in-place/13046202>

<https://www.abc.net.au/news/2021-03-29/sa-imposes-hard-border-on-greater-brisbane/100035588>

VIC

<https://www.dhhs.vic.gov.au/coronavirus-update-victoria-sunday-11-october>

<https://www.dhhs.vic.gov.au/updates/coronavirus-covid-19/face-coverings-mandatory-melbourne-and-mitchell-shire>

<https://www.coronavirus.vic.gov.au/face-masks-when-wear-face-mask>

<https://www.premier.vic.gov.au/statement-premier-63>

[https://www.premier.vic.gov.au/atement-premie](https://www.premier.vic.gov.au/atement-premier)

<https://www.premier.vic.gov.au/state-emergency-coronavirus-extended-save-lives>

<https://www.premier.vic.gov.au/state-emergency-extended-keep-slowing-spread>

<https://www.premier.vic.gov.au/state-emergency-extended-keep-slowing-spread> <https://www.premier.vic.gov.au/statement-premier-72>

<https://www.9news.com.au/national/coronavirus-melbourne-holiday-inn-cluster-grows-fears-of-another-lockdown-third-wave/76487987-e76d-4868-9949-71345bf083bf>

TAS

<https://www.cotatas.org.au/news-items/coronavirus-covid-19-advice/#stage2>

<https://www.cotatas.org.au/news-items/coronavirus-covid-19-advice/#stage2>

<http://www.premier.tas.gov.au/releases/tasmanias_roadmap_to_recovery>

<https://www.health.tas.gov.au/news/2020/Important_information_about_new_COVID-19_response_measures>
