# Supplementary material for: Living with a genetic, undiagnosed or rare disease: A longitudinal journalling study through the COVID‐19 pandemic
Source: Health Expect. 2022 Feb 5;25(5):2223–34. doi: 10.1111/hex.13405 (PMC9111564; doi:10.1111/hex.13405)
Supplement: Supplementary file 2 — Supporting information. [file HEX-25--s002.docx]

### **
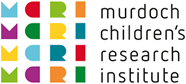

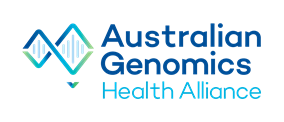
**
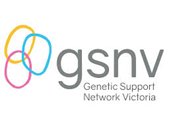


**COVID19 Journals: The lived experience of the Genetic, Undiagnosed and Rare Disease Community**

Thank you for signing up to the COVID-19 Journals: The lived experience of the Genetic, Undiagnosed and Rare Disease Community study. We are excited to have you on board. The Participant Information Sheet gave you an outline of the study and here is a reminder and some more detail. Remember you can contact the study leads at any time.

**What should I be recording?**

You can record anything you want! We are interested in what has changed for you, how are you working your way round any restrictions in place due to COVID-19, what changes have come into place that work for you – and maybe you didn’t expect them to(!) – what would you like to see continue on into the future.

**How should I be recording my journal?**

Any way you wish, it may be written, such as in a Word document, a journal app (there are lots of free ones online) or even handwritten. The journal can be in any format you prefer, for example, text, photographs, creative contributions such as pictures, to share your ongoing lived experiences of the current turbulent environment and as we come out the other side. Remember, if you take photographs, you must have the consent of anyone who is in them. It would be helpful for us to know what the artwork/photograph represents.

Some people like structure. If that’s you it may be helpful to think about an event (positive or negative) that you experience, think about what that experience means or meant to you and what you have learnt from the experience.

**What about risks, side effects, discomforts and inconveniences?**

Many people enjoy keeping a journal but for some it may be upsetting to be reflecting on what is going on in their lives. Help is available and here are somethings you can do:

- Talk with your friends and family;
- Contact any of the genetic peer support groups, including the GSNV. They hold a Thursday morning phone in (10.30-12.00 <https://gotomeet.me/gsnv>);
- Go to the Beyond Blue website (<https://www.beyondblue.org.au/> ) who also have online and phone support available;
- Remember your GP is always available for you to talk to.

**How often will you ask for my journal?**

We will email you once a month over the next year to ask you to share whatever journal entries you are happy to share.

**Can I stop taking part?**

Yes – anytime. If you wish to withdraw from the study just email [c19journals@mcri.edu.au](mailto:c19journals@mcri.edu.au) and we will take you off the email list. You won’t receive any more reminders.
