# Supplementary material for: Living with a genetic, undiagnosed or rare disease: A longitudinal journalling study through the COVID‐19 pandemic
Source: Health Expect. 2022 Feb 5;25(5):2223–34. doi: 10.1111/hex.13405 (PMC9111564; doi:10.1111/hex.13405)
Supplement: Supplementary file 3 — Supporting information. [file HEX-25--s003.docx]

**Supplementary file**

Table S3: Quotes from journals representing the Resilience Scale for Adults and Thematic Analysis - additional examples

| **RSA Code** | **Examples** |
| --- | --- |
| Structured style | "Every day starts with a check of the diary. With predictable routines and a diary of planned activities, [Shane] is able to function well and have a sense of control of his life, so very important for someone who is deafblind" (Participant 1, carer/support sector, age 46-59, VIC, July 2020)  “I do the dishes most mornings and help hang out the washing. Once or twice a week I’ll vacuum and mop the floors. I make sure to eat meals/snacks at about the same time each day, prioritise sleep and do something I enjoy doing every day.” (Participant 21, individual, age 18-29, QLD, September 2020) |
| The perception of self | "Once again, I feel no motivation (feel depressed) so I decided to go back on the dexamphetamine, but only half a tablet to reduce the jittery side effects." (Participant 27, individual/carer/support sector, age 46-59, VIC, February 2021)  "I’m just so downtrodden. As if it hasn’t been a massive fight to get this good, only to throw it all away? It would be different if I was relapsing, but I’d like to think I’m excelling. Not necessarily in the traditional sense of “success” but in terms of my own values." (Participant 21, individual, age 18-29, QLD, November 2020) |
| Family cohesion | "The mental health battles for all in the family are real and we all have strategies in place and are making headway." (Participant 8, carer, age 46-59, VIC, September 2020)  "But I feel like we are going to be stuck like this forever. I just want to leave and go back to Cairns. But I can’t because they just won’t let me in. And my daughter just wouldn’t get the right medical care back in Cairns. I’m trying very hard to be patient and positive, to keep everyone in my household happy and positive. But it’s a struggle." (Participant 27, individual/carer/support sector, age 46-59, VIC, October 2020) |
| Social resources | "We had one support person that would care for our daughter when our son was in hospital" (Participant 7, carer, age 30-45, VIC, July 2020)  "It is so good being able to ring [the brain rehab case worker] at any time and run things by her, helps to get my thoughts in order and workout how to go forward. So lucky to have her." (Participant 2, carer/support sector, age 46-59, New South Wales, August 2020) |
| Social competence | "I have been able to meet new people through the online world who I wouldn't otherwise have known, and I am thankful for these opportunities." (Participant 23, individual/support sector, age 18-29, VIC, September 2020)  "I am going out of the house more and getting more social with friends and family. It has been so much better without the tight restrictions." (Participant 6, individual, age 18-29, VIC, November 2020) |
| Direct impact of Covid-19 on health and social care services | "The hospital visiting hours were now severely restricted due to Covid… The Covid 19 pandemic has seen new VERY STRICT rules introduced to hospitals." (Participant 27, VIC, Individual/Carer/Support Sector, age 46-59, July 2020)  "[Marvin] had a seating assessment (wheelchair) and full Occupational Therapy assessment by OT from MSWA. Due to Covid there are long waiting lists but we were able to have the assessment prioritised within a week. There is currently a 2-3 month wait." (Participant 28, WA, Carer, age 60-75, March 2021) |
| Turning Points | "Our Zoom meet ups are finally organised! Better late than never we have had many training sessions, peer training sessions, peer-support training will be great for our members I think, this is going to establish permanent infrastructure in our group, utilising our online resources and the people themselves to a level that we haven’t before. We had our first ‘Zoominar’ as opposed to a meet up" (Participant 20, QLD, Individual/Support Sector, age 18-29, September 2020)  "Announcement of lockdown lifting" (Participant 14, VIC, Individual, age 18-29, October 2020) |
| Coping strategies | "I’ve been watching a lot of ASMR-style soap carving or sand cutting videos, not because I experience that sensation but because, when my mind is so thick and foggy, it’s the only kind of entertainment I can focus on (as well as being soothing)." (Participant 21, QLD, Individual, age 18-29, February 2021)  "We need to go back to brain injury basics of phone reminders, calendars, journals etc for her" (Participant 2, NSW, Carer/Support Sector, age 46-59, February 2021) |
